# Descriptive study of measles vaccination second dose reporting and barriers to improving coverage in six districts in Malawi

**DOI:** 10.11604/pamj.supp.2020.35.1.19096

**Published:** 2020-01-03

**Authors:** Geoffrey Chirwa, Karen Annette Wilkins, David John Mercer

**Affiliations:** 1 Ministry of Health, Malawi; 2 Orlando St SW, Atlanta, Georgia 30311, USA

**Keywords:** Measles vaccination, measles elimination, measles second dose, data quality audit, data quality assessment, vaccination coverage

## Abstract

**Introduction:**

Malawi’s National Immunization Program introduced a second routine dose of measles containing vaccine (MCV2) in 2015 but found coverage lagging. We assessed data quality and gaps in service delivery.

**Methods:**

Investigators used a modified data quality audit in 6 low performing districts accompanied by questionnaires for health facilities (HF) and households with children with >1 vaccination.

**Results:**

MCV2 doses administered according to source were: 733 in registers, 2364 in reports, 1655 in district reports, 2761 in the electronic database. There was 77% agreement regarding status for MCV2 between the register and the home-based record (HBR). Drop-out differences were found between HF according to the practice of waiting for a minimum number of children to open an MCV vial, canceling sessions due to stock-out and requesting payment for a home-based record. Eighty one percent (81%) of children whose caregivers knew 2 doses were needed had received MCV2 vs fifty eight (58%) of children whose caregivers didn’t know. Sixty two (62%) of children who were charged for HBR received MCV2 vs 78% reporting no charge.

**Conclusion:**

The drop-out between the first and second doses of MCV was high and inconsistent with elimination goals. The quality of administrative data in these 6 districts was found to be poor. This investigation found that session cancelation, charging for HBR and lack of caregiver knowledge affected completion of the vaccination series. The authors recommend program improvements in these areas to increase uptake of MCV2 and improved reporting practices at all levels of the system.

## Introduction

Measles is a highly contagious disease that prior to widespread vaccination killed an estimate of 2.6 million globally every year [1]. With the introduction of an effective measles vaccine and routine coverage levels over 80%, elimination is considered feasible and a strategic plan to achieve that goal has been developed by the Measles and Rubella Initiative (MRI). In the 2012-2020 plan, MRI calls for countries to “achieve and maintain high levels of population immunity by providing high vaccination coverage with two doses of measles- and rubella-containing vaccines” and sets a target of 95% coverage with a first and second dose of measles-containing vaccine (MCV1 and MCV2) in each district and every country. In 2011, countries in the African Region of WHO adopted the goal to eliminate measles by 2020 [2]. Vaccination against measles started as a routine program in Malawi in 1980 with one dose given at 9 months of age. Coverage as a percent of children under one year of age vaccinated steadily increased reaching 89% in 2017 [3]. Malawi also conducted 7 national Supplementary measles immunization activities (SIAs) from 2000 to 2017 targeting age ranges defined by the epidemiology of the disease at the time. As a result of routine vaccination and SIAs, the reported number of cases of measles steeply declined from 1989 through 2009 [3]. A large measles outbreak in 2010 as well as a WHO recommendation [4] led the Ministry of Health, National Immunization Program to adopt a second routine dose of measles containing vaccine (MCV2) into the schedule at 15 months of age [5]. The program introduced MCV2 progressively in all districts from July through December 2015. An MCV2 post introduction evaluation (PIE) conducted in October 2016 found that MCV2 coverage for the period January - June 2016 was 57% [6], lagging behind MCV1 coverage and insufficient to reach measles elimination goals [2]. This is consistent with coverage levels of several other countries in the African region as documented by Masresha et al. [7]. However, the PIE was unable to determine if the low coverage was due to poor reporting or to service delivery challenges, leading the Ministry of Health to conduct an additional investigation in February 2017. The objectives of this descriptive investigation were to: a) determine if records of MCV1 and MCV2 doses administered in health facility immunization registers agree with the numbers reported to national level; b) determine if record of MCV2 in the health facility immunization register agrees with the home-based record; c) identify non-data gaps in service delivery that might contribute to non-completion of vaccination among children who began the series.

## Methods

Six of the country’s 29 districts were chosen based on low MCV2 coverage for the period January - June 2016 as reported through the administrative reporting system plus additional districts chosen to provide an urban/rural mix. Within each district, a simple random sample of 3 health facilities (HF) was chosen. Using a modified data quality audit methodology [8], the number of doses of tracer vaccinations (MCV1 and pentavalent (diphtheria, pertussis, tetanus, hemophilus influenzea type B, hepatitis B) vaccine administered to children less than 12 months and more than 12 months of age, and MCV2) was collected from immunization registers, tally sheets and monthly reports at the facility and districts level; and from electronic databases for those facilities at the district and national levels for the months of February, October and December 2016 using standardized tools. A standardized questionnaire covering vaccination practice was administered to Health Surveillance Aids (HSA) in each HF.

To identify children who had begun their primary vaccination series, a simple random sample of 2 communities per HF was chosen by the interviewers using the facility immunization register. All children: a) from those communities; b) born between December 1, 2014 and August 30, 2015 and c) who received at least one vaccination between January 1 and October 31, 2015 were listed and 6 systematically selected for household visits. If there were less than 6 children from a chosen community, a neighboring community was selected based on local knowledge of geography. Households were visited and care givers of children identified from the register were interviewed using a standardized questionnaire and the information in the home-based vaccination record was copied. For purposes of comparing HF, drop-out between MCV1 and MCV2 was calculated from household questionnaires and HF divided into higher and lower drop-out in relation to the mean rate. One HF for which household questionnaires were not completed was excluded from this analysis. Data were collected using tablets preloaded with questionnaires in ODK and analyzed using MSExcel and EpiInfo.

## Results

Information was successfully collected at the national level, in all 6 districts and 18 health facilities. After deduplication, there were 189 child records from the immunization registers of 17/18 health facilities and from 38 communities. The immunization register was not useable for child identification in the 18th HF. The health facilities interviewed were 2 hospitals, 12 health centers, 2 dispensaries, 1 clinic and 1 health post. Of the 189 children sought from the register, there was 1 death, 1 refusal and 24 who were not located. Of the 163 interviews, four children no longer lived in the location in the register resulting in 159 completed household questionnaires. The age of the children included in the sample provided a range from 17 through 26 months of age at the time of the survey. There were 6 to 14 household questionnaires per health facility.

### Data quality

**Figure 1:**
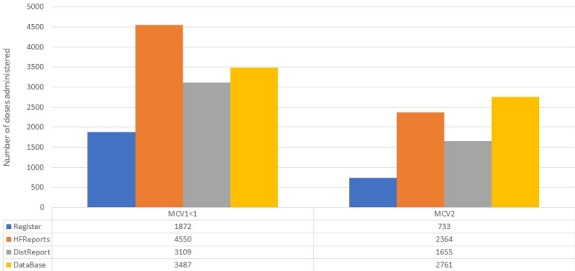
Total doses MCV1 < 1 and MCV2 administered by source, 18 health facilities

Immunization registers were available in all health facilities visited. Tally sheets were present and being used in 1/18 HF. Completed monthly reports were found for all 3 months in 17 HF, in the 18th HF 2 completed forms were located. Monthly report forms with printed space for MCV2 were available in 17/18 HF. 7/18 HF reported stockouts of reporting forms for the monthly report and or vaccination cards in 2016 ranging from 1 to 6 months with an average of 3 months. The number of doses recorded administered were collected from HF. As can be seen in Table 1, a total of 1872 doses of MCV1 administered were found in immunization registers and 4550 found in monthly reports. For MCV2, a total of 733 doses administered were found in immunization registers and 2364 found in monthly reports. A total of 4550 doses MCV1 administered were found in health facility monthly reports located at the district level and 3109 found in monthly reports prepared by the district with 10 exactly agreeing. A total of 2362 doses MCV2 administered were found in HF monthly reports located at the district and 1655 found in monthly reports prepared by the district level with 8 exact matches. A total of 3109 doses MCV1 were found in the monthly reports for the health facilities prepared by the district and 3487 in the electronic database at the national level for the 18 health facilities. For MCV2, a total of 1655 doses administered were reported per monthly reports and 2761 per the electronic database. The differences between total numbers of doses administered by source is illustrated in Figure 1. There was 76% and 77% agreement regarding vaccination status for MCV1 and MCV2 respectively between the register and the home-based record (HBR) without taking dates into consideration (Table 2). More children were vaccinated per HBR (128 vs. 97 MCV1; 78 vs. 70 MCV2) than per the immunization register.

**Table 1:** Doses of Measles containing vaccine (MCV) administered by Health Facility and by information source, February, October and December 2016

HF #	HF level				District level				National			
	MCV1 <1		MCV2		MCV1 <1		MCV2		MCV1 <1		MCV2	
	*Register*	*Monthly reports*	*Register*	*Monthly report*	*Monthly report from HF*	*Monthly report of District*	*Monthly report from HF*	*Monthly report of District*	*District Monthly report*	*Electronic database*	*District Monthly report*	*Electronic database*
1	155	816	38	1	816	564	1	2	564	578	2	283
2	29	62	35	46	62	77	46	46	77	99	46	117
3	40	75	24	1	75	69	1	19	69	60	19	51
4	452	1709	0	1180	1709	546	1180	480	546	1470	480	1180
5	144	382	0	148	382	382	148	148	382	321	148	258
6	98	100	81	170	100	100	170	170	100	89	170	170
7	261	325	121	195	325	315	195	194	315	94	194	96
8	79	120	0	40	120	120	40	40	120	100	40	40
9	70	102	18	26	102	74	26	26	74	63	26	26
10	270	276	158	148	276	276	148	148	276	198	148	148
11	72	185	61	150	185	187	150	150	187	131	150	150
12	50	62	41	60	62	62	60	39	62	70	39	66
13	29	109	35	71	109	109	71	72	109	50	72	72
14	29	59	28	32	59	59	32	34	59	47	34	34
15	19	13	8	12	13	13	12	5	13	13	5	5
16	30	107	32	38	107	107	38	34	107	64	34	29
17	14	23	22	26	23	24	26	28	24	17	28	16
18	31	25	31	20	25	25	20	20	25	23	20	20
Total	1872	4550	733	2364	4550	3109	2364	1655	3109	3487	1655	2761

HF4: one monthly report not found at the HF level

**Table 2:** Vaccination status by source of information: home-based record and immunization register

	MCV1			MCV2		
From Home- based record	From immunization register			From immunization register		
	Yes	No	Total	Yes	No	Total
Yes	96	32	128	58	20	78
No	1	11	12	12	50	62
Total	97	43	140	70	70	140

### Factors affecting MCV2 coverage

The 17 HF with household questionnaires were compared for factors that might influence drop-out (Table 3). Health facilities with less than average drop-out rates planned an average of 8.7 static and 4.6 outreach under-2 well child clinics per month and offered MCV at all sessions. Seventy eight (78%) reported that they had no minimum number of children required to be present to open a vial of measles vaccine. Twenty-two percent reported that they had canceled at least one vaccination session due to stock-out of vaccine. This question was not specific to measles vaccine. Eleven percent stated that they prioritized who would receive measles vaccine if stocks were low. Eleven percent reported that they requested payment for the home-based record, none requested payment for services. The 8 health facilities with greater than average drop-out planned an average of 11.6 static and 3.1 outreach under-2 well child clinics per month, MCV was offered at 87.5% of static and 100% of outreach sessions. All reported that they had no minimum number of children to open a vial of measles vaccine. Sixty-two percent had canceled at least one vaccination session due to stock-out of vaccine. This question was not specific to measles vaccine. Zero percent stated that they prioritized who would receive measles vaccine if stocks were low. Sixty-two percent reported that they requested payment for the home-based record, none requested payment for services.

**Table 3:** Selected reported service delivery behavior for Health Facilities with less than and greater than average drop-out (DO) rate

		% of HF	
		<30% DO (n=9)	≥30% DO (n=8)
Number sessions/month	Static	8.7	11.6
	Outreach	4.6	3.1
Vaccinate every session	Static	100	100
	Outreach	100	100
Offer MCV every session	Static	100	87.5
	Outreach	100	100
No min children to open MCV Vial		78	100
Canceled at least one session due to stockout		22	62.5
Prioritize for MCV if stock is low		11	0
Request payment for Vax card		11	62.5
Request payment for session		0	0

Among the children included in the household survey, 9 MCV1 vaccinations were misrepresented as MCV2 (in the absence of a first dose, older than one year at the time of vaccination). These were corrected to MCV1. 146/159 children (91%) had a home-based vaccination record present at the time of the survey, 143 of these were the government-issued passport and 3 a vaccination card or other record. Vaccination status was estimated from the home-based record and parental recall. 150/159 (94%) of children had received MCV1 and 106/159 (67%) MCV2 based on the home-based record or care-giver recall - a drop-out rate of 29% (Table 4). Sixty-two percent of respondents thought that the child had received all of his/her vaccinations, 71% of whom had received MCV2. Ninety-eight percent of respondents had heard of measles vaccination. Forty-three percent knew that a child needed 2 doses of MCV. Of those that said a child needed 2 doses, 81% had received MCV2. Of the 53% (85/159) who thought the child needed one dose or said that they did not know 58% had received MVC2. New HBR were printed for the introduction of MCV2 but not all children were in possession of the new cards. Sixty-eight percent of those that had the new cards with MCV2 pre-printed had received that dose. Sixty-seven percent of those with the HBR without MCV2 had received that dose. Of those who reported that they paid for the HBR, 62% had received MCV2. Seventy-eight percent who reported they had not paid for HBR had received MCV2 (Table 4). Eighty-nine percent of caregivers said that the health care worker (HCW) was the most trusted source of information regarding vaccination and 79% that they knew when to return for a vaccination because the HCW had told them during the previous clinic visit.

**Table 4:** Vaccination coverage by source and selected care-giver responses by vaccination status, MCV2 (card + history)

	MCV1 (n=159)	MCV2 (n=159)		
	Number	%	Number	%
Card	123	77	90	57
History	8	5	8	5
Total vaccinated	131	82	98	62
		**MCV2 (card + history)**		
		**Yes**	**No**	**% Vaccinated**
Is your child completely vaccinated?	Yes	99	40	71
	No	5	10	33
	don’t know	2	3	40
How many doses does a child need?	1	23	18	56
	2	56	13	81
	don’t know	26	18	59
MSD pre-printed on card	Yes	27	13	68
	No	71	35	67
Paid for home-based record	Yes	63	38	62
	No	35	10	78
Stay with other adult	Yes	66	34	66
	No	40	19	68
Due for MCV1 before MCV2 intro		48	24	67
Due for MCV1 after MCV2 intro		47	27	64

## Discussion

We confirmed that drop-out between the first and second doses of MCV is a problem in the 6 districts included the study. Drop-out ranged from 21% to 61% depending on the data source used, all higher than levels needed to achieve the measles elimination goal endorsed by the national, regional and global levels. This study determined that records of MCV1 and MCV2 doses administered were inconsistent from HBR to HF registers to HF monthly reports, through the district level reports to the national database. This lack of consistency undermines the confidence of program managers at all levels. The figures available at district and national levels were 1.7 - 2.4 times higher than those found in immunization registries, masking the probable low coverage in the HF and the high drop-out rates limiting the use of the data for planning or evaluation. The immunization register at the HF is a key tool in identifying children who have missed doses for follow-up and, in the absence of tally sheets, is the only source of information for the monthly reports [9]. In one of these HF, the immunization register was not name-based, making defaulter tracking impossible. In the other 17, immunization registers missed 25% of the MCV1 doses and 26% of the MCV2 doses found in HBR.

This investigation was not designed to provide a coverage estimate for the districts included. Based on the design, which was intended to determine the quality of reported information and identify potential factors contributing to high drop-out between MCV1 and MCV2, only children who had initiated the vaccination series and were identifiable from the immunization registry were included. A variable number of households were interviewed by HF further limiting interpretation of the responses. Therefore, results of the household component of the investigation are not representative of the HF nor the districts but have provided hypotheses for high drop-out which can be further tested.

While not representative, this investigation found that drop-out was higher than average in HF that more frequently reported cancellation of at least one vaccination session due to stock-out and who reported they charged for HBR. The household questionnaires also identified care giver reports of paying for the HBR as potentially resulting in less MCV2 coverage. The HBR is an essential tool for reminding care givers of vaccination history and facilitating screening for vaccinations due during immunization sessions or curative care [9, 10]. The Malawi national policy is consistent with WHO recommendations and common practice [11]. However, this investigation demonstrates that the policy is not universally followed and may have a negative impact on completion of the vaccination series. Efforts must be undertaken to assure the respect of national policy and provision of HBR free of charge.

Caregiver knowledge of the need for a second routine dose of measles vaccine was another factor identified as potentially affecting MCV2 coverage with 81% of those who knew receiving MCV2 vs 58% of those who thought one dose was needed or replied that they did not know. While interpretation of this observation is further limited by directionality (mothers of children who received 2 doses are more likely to know about the need for 2 doses), HCW are the most trusted source of information regarding immunization in general and the next appointment in particular. HCW must increase communication about the need for a second dose of MCV.

## Conclusion

In summary, we found that the drop-out rate between the first and second doses of MCV was high and inconsistent with measles elimination goals. We also found that the quality of administrative data in these 6 districts was poor and greater attention needs to be paid at all levels to improve the collection and use of data. Additionally, this investigation found that session cancelation, charging for HBR and lack of caregiver knowledge are potential factors affecting completion of the vaccination series. These hypotheses may be tested through additional studies. Authors recommend undertaking program improvements that focus on these areas to increase uptake of MCV2 and improve reporting practices at all levels of the system are logical and recommended. Following this investigation, the immunization program began implementation of missed opportunity for vaccination [12] and second year of life [13] strategies in 3 districts.

### What is known about this topic

- Coverage with a second routine dose of measles containing vaccine lags behind coverage with the first dose in many countries, including Malawi;
- Health worker behavior does not always follow national policies.

### What this study adds

- Addressing the challenge of low coverage and high drop-out between measles doses is complicated by poor data quality;
- Despite national policy on providing home-based-records free of charge, some HF continue to charge which seems to have an effect on completing MCV2;
- Frequent cancelation of sessions due to stock-out may have an effect on drop-out rates.

## Competing interests

The authors declare no competing interests.
